# Amygdala volume and psychiatric burden in focal epilepsy: PET evidence for a glial substrate

**DOI:** 10.1093/braincomms/fcag394

**Published:** 2026-10-16

**Authors:** Theodore P Imhoff-Smith, Alan McMillan, Samuel A Hurley, Mariel Kalkach Aparicio, Veena Nair, Santiago Philibert-Rosas, Jeffrey R Binder, Mary E Meyerand, Jana Jones, Bruce Hermann, Vivek Prabhakaran, Nagesh Adluru, Aaron F Struck

**Affiliations:** Department of Radiology, University of Wisconsin-Madison, Madison, WI 53792, USA; Department of Radiology, University of Wisconsin-Madison, Madison, WI 53792, USA; Department of Radiology, University of Wisconsin-Madison, Madison, WI 53792, USA; Department of Neurology, University of South Dakota, Sioux Falls, SD 57105, USA; Department of Radiology, University of Wisconsin-Madison, Madison, WI 53792, USA; Department of Neurology, Washington University in St. Louis, St. Louis, MO 63110, USA; Department of Neurology, Medical College of Wisconsin, Milwaukee, WI 53226, USA; Department of Medical Physics, University of Wisconsin-Madison, Madison, WI 53705, USA; Department of Neurology, University of Wisconsin-Madison, Madison, WI 53705, USA; Department of Neurology, University of Wisconsin-Madison, Madison, WI 53705, USA; Department of Radiology, University of Wisconsin-Madison, Madison, WI 53792, USA; Department of Medical Physics, University of Wisconsin-Madison, Madison, WI 53705, USA; Department of Neurology, University of Wisconsin-Madison, Madison, WI 53705, USA; Department of Radiology, University of Wisconsin-Madison, Madison, WI 53792, USA; Waisman Center, University of Wisconsin-Madison, Madison, WI 53705, USA; Department of Neurology, Washington University in St. Louis, St. Louis, MO 63110, USA

**Keywords:** glia, neuroinflammation, TSPO, MRI-negative

## Abstract

Temporal lobe epilepsy is of heterogeneous pathology. While many patients present with hippocampal sclerosis and ipsilateral mesiotemporal atrophy, a subgroup of patients without human-detectable sclerosis via structural MRI are characterized by ipsilateral amygdala enlargement and worse postsurgical outcomes. A bidirectional relationship between temporal lobe epilepsy and depression is well-established, with amygdala enlargement and neuroimmune alterations increasingly implicated as substrates for both. In this study, we interrogate the link between amygdala volume and psychiatric burden in temporal lobe epilepsy and evaluate gliosis as a possible substrate. We hypothesize that gliosis drives ipsilateral amygdala volume changes that underlie psychiatric burden in patients with no hippocampal sclerosis. To test these relationships, we analyzed two independent cross-sectional cohorts: (i) a Discovery cohort (43 patients, 80 controls) with structural MRI and robust psychiatric measures and (ii) a Mechanistic pilot cohort (10 patients, 9 controls) with both structural MRI and [^18^F]FEPPA PET/MR, an in vivo measure of gliosis, to conceptually replicate and extend findings from the Discovery cohort. Amygdala volumes were assessed with FreeSurfer. Psychiatric burden in the Discovery cohort was assessed with the Depressive Problems subscale from the Achenbach Adult Self-Report. Regional gliosis in the Mechanistic cohort was quantified using [^18^F]FEPPA PET, and an exploratory measure of psychiatric burden for this cohort was obtained via medical chart review. In the Discovery cohort, the group without hippocampal sclerosis showed significant ipsilateral enlargement of the whole amygdala, basolateral and central subnuclei compared to controls and the group with sclerosis and a group-dependent association between decreased amygdala volume and increased depressive symptoms. In the Mechanistic cohort, increased [^18^F]FEPPA binding was associated with reduced amygdala volume. Adjusting for this effect unmasked significant amygdala enlargement in the group without hippocampal sclerosis. Exploratory analysis revealed a possible central subnucleus-specific relationship between gliosis, volume and psychiatric burden in patients without sclerosis, such that amygdala volume was positively correlated with psychiatric burden specifically in patients with a greater degree of gliosis. These data associate amygdala volume alterations with psychiatric burden in patients without sclerosis and implicate gliosis as a potential mechanistic substrate. Findings support a conceptual shift towards understanding amygdala enlargement not as a static structural phenotype, but as a dynamic, glia-associated state that may confer risk for psychiatric illness. Consequently, patients without hippocampal sclerosis may derive a distinct dual benefit from therapeutic strategies targeted at underlying neuroimmune function.

## Teaser

Using simultaneous PET/MR, Imhoff-Smith *et al*. show that, rather than a static structural phenotype of focal epilepsy, amygdala volume alterations reflect a possibly dynamic, glia-associated state linked to psychiatric burden. Converging evidence from this multimodal analysis offers a novel framework to reconcile conflicting reports of amygdala enlargement and psychiatric risk.

## Graphical Abstract

**Graphical Abstract:**
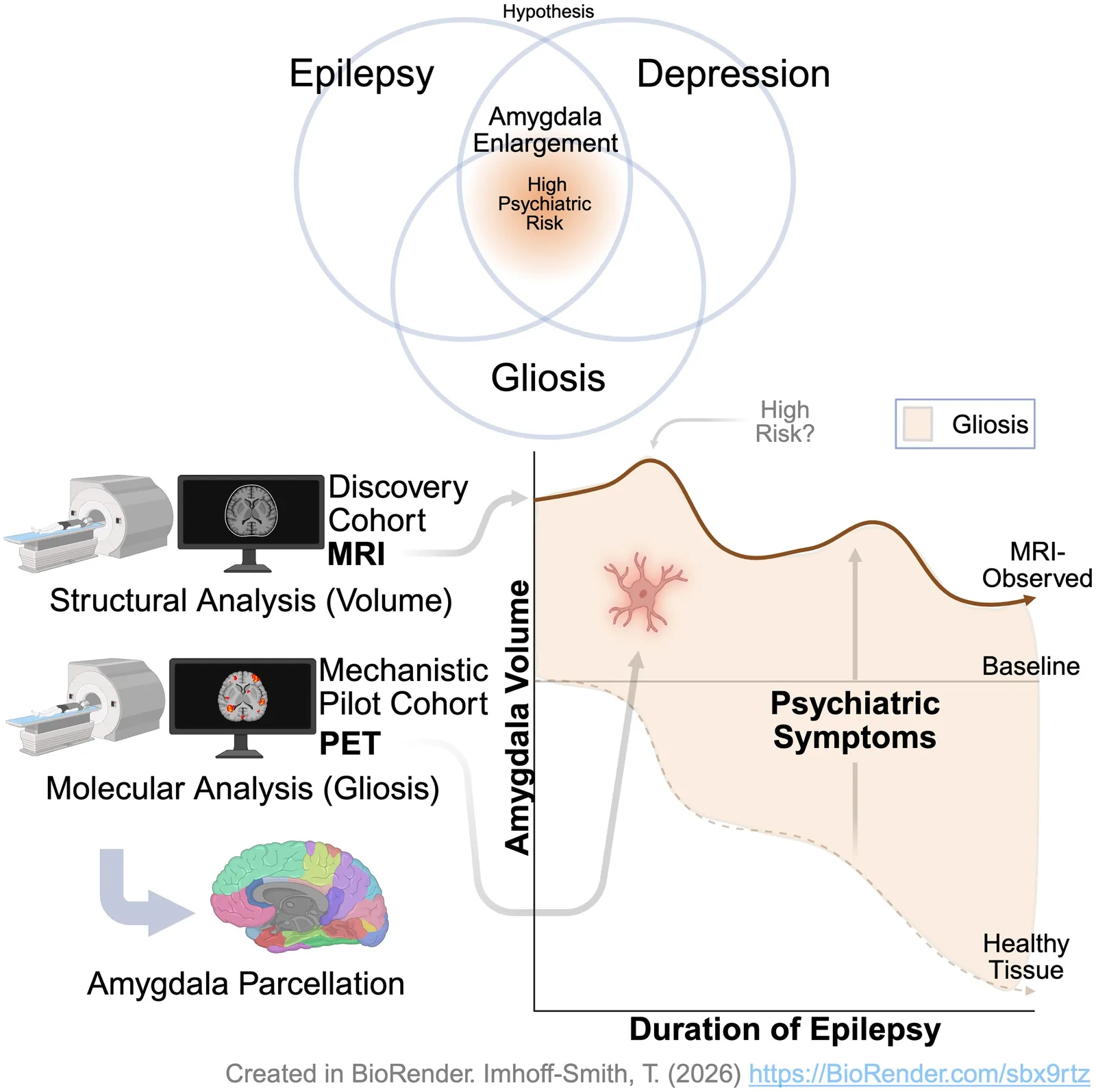

## Introduction

Temporal lobe epilepsy (TLE) is a heterogeneous disorder often characterized by hippocampal atrophy and glial fibrillary scarring known as hippocampal sclerosis (HS).^1^ However, roughly 40% of patients do not exhibit human-detectable sclerotic lesions via structural MRI.^2,3^ The amygdala is also a critical pathological site that often distinguishes these two groups. Both reduced ipsilateral amygdala volume^4^ and contralateral amygdala enlargement (AE)^5^ have been reported in patients with HS; conversely, patients without HS (no-HS) often exhibit ipsilateral AE,^6-10^ a feature virtually absent in patients with HS.^11^ These no-HS patients are characterized by worse rates of postsurgical seizure freedom and progressive cognitive decline, making treatment of this subgroup a particular challenge.

Amygdala enlargement has also been reported in the primary depression literature.^12-14^ Given the well-documented bidirectional relationship between depression and epilepsy,^15,16^ AE may therefore serve as a transdiagnostic marker of a shared underlying pathophysiology. Yet, if enlargement is directly linked with depression, reduced ipsilateral amygdala volume in patients with HS presents a paradox, particularly given that the prevalence of mood disorders in this group is comparable to that of patients without HS.^17^ Moreover, while the relationship between amygdala volume and depression has been studied most extensively, reports of AE in TLE are not exclusive to depression,^18,19^ and some evidence suggests that the link between AE and psychiatric outcomes may even be isolated to enlargement of specific subnuclei.^20^ Taken together, these reports suggest a possibly distinct neurobiological substrate that modulates psychiatric burden beyond the macroscopic pathologies targeted by surgery.

Gliosis is a prime suspect in both epilepsy-comorbid depression^21^ and no-HS epilepsy, with as many as two-thirds of patients with no-HS exhibiting histologically confirmed gliosis.^6,22^ Altered immune pathways^23^ and distinct astrocyte morphology have been described in patients with no-HS, leading to the proposal of an ‘innate inflammatory gliosis-only’ (I^2^GO) subtype of TLE.^7^ In parallel to the strikingly high psychiatric burden in epilepsy—where nearly 70% of patients with pharmacoresistant TLE experience depression and/or anxiety symptoms^24,25^—a pharmacoresistant inflammatory subtype of depression has also emerged.^26,27^ Concordantly, glial dysfunction during the immune response may be one potential pathway driving elevated risk for psychiatric illness.^28^ However, the aetiology of AE remains under debate. While some evidence suggests a possible link between AE and gliosis,^7,8^ histopathological assessment of the enlarged amygdala does not always identify gliosis,^29^ and the specific link between AE and psychiatric burden is not well understood. Recent advances in molecular imaging have enabled more precise quantitation of *in vivo* glial activity through second-generation translocator protein radioligands, such as [^18^F]FEPPA,^30,31^ yet translational studies of glial contributions to clinical manifestations of epilepsy are limited.

We hypothesized that ipsilateral AE would correlate with psychiatric burden specifically in patients without HS, and that AE would be associated with amygdala gliosis. Consequently, in this study we test for an HS-dependent link between gliosis, AE and psychiatric burden. Using a Discovery cohort (*N* = 123) from the Epilepsy Connectome Project, we compare amygdala volume differences between no-HS, HS and typical controls, and test the link between these volumetric differences and depressive symptoms. We use an independent Mechanistic pilot cohort (*N* = 19) with [^18^F]FEPPA PET/MR (enabling simultaneous measurement of gliosis and amygdala volume) to conceptually replicate and extend AE findings from the Discovery cohort by assessing the effect of gliosis on differences in amygdala volume. Finally, we conduct an exploratory analysis testing the moderating effect of gliosis on the link between volume alterations and psychiatric burden in patients without HS. We evaluate subnuclear specificity of these relationships throughout.

## Materials and methods

### Discovery cohort

#### Participants and recruitment

Patients with TLE and controls who participated in the Epilepsy Connectome Project, a two-site research project involving the Medical College of Wisconsin and the University of Wisconsin-Madison, were retrospectively recruited.^32,33^ Eligible participants were 18–60 years old, and patients had a diagnosis of TLE supported by two or more of the following: (i) described or observed clinical semiology consistent with seizures of a temporal lobe origin, (ii) EEG evidence of either Temporal Intermittent Rhythmic Delta Activity or temporal lobe epileptiform discharges, (iii) temporal onset of seizures captured on EEG and (iv) MRI evidence of mesial temporal sclerosis or hippocampal atrophy. Initial recruitment also excluded patients with any of the following: (i) Presence of epileptogenic lesions other than mesial temporal sclerosis and non-specific white matter abnormalities on 3 Tesla (3T) MRI, (ii) active infectious aetiology of seizures, (iii) suspected or confirmed evidence of active autoimmune or inflammatory process in the central nervous system. Participants underwent behavioural testing and anatomical scans were obtained during visits at either the University of Wisconsin-Madison or the Medical College of Wisconsin. All participants gave written informed consent. All procedures were reviewed and approved by the Institutional Review Board at the Medical College of Wisconsin. Demographics, self-reported symptoms and T1-weighted (T1w) imaging for this Discovery cohort were subsequently acquired from the publicly accessible repository via Open Science Framework (https://osf.io/exbt4/). Patients were further screened for inclusion in this study if they also met the following criteria: available T1w MRI, unilateral seizure onset zone and available data for the presence of HS (Supplementary Fig. 1).

#### Procedures

##### Psychiatric measurement

Given that the relationship between AE and depression is most extensively characterized in existing literature, we focused our primary analysis in the Discovery cohort on depressive symptoms. These were quantified using the Achenbach Adult Self-Report (ASR) Depressive Problems subscale for patients and controls. This instrument provided a well-validated measure of depressive symptoms for foundational analysis of the ‘group’ by ‘volume’ effect on psychiatric burden.^34^

##### Anatomical MRI processing

High-resolution anatomical images were acquired on standard 3T GE Healthcare Discovery MR750 MRI systems with a 32-channel head coil using a 3D MPRAGE sequence (TR/TE/TI: 604/2.516/1060 ms; flip angle: 8°; field of view [FOV]: 25.6 cm; 0.8 mm isotropic). Images were processed using FreeSurfer (version 7.4.1) to reconstruct and derive anatomical parcellations using the Desikan–Killiany Atlas and FreeSurfer subcortical segmentation. Visual quality control was conducted to ensure the absence of gross artefacts from anatomical segmentations. To enable comparison across the full sample, imaging metrics were extracted, and patient amygdala volumes were aligned to a common hemisphere (ipsilateral versus contralateral to the side of the seizure onset zone). Bilateral mean was calculated for controls to provide a normative baseline. Amygdala subnuclear volumes were combined into four regions of interest (ROIs) defined by prior literature.^4,35,36^ Each ROI was subsequently normalized by estimated total intracranial volume (ICV), forming basolateral (BLA; basal, lateral, accessory basal and paralaminar), central (CeA), medial (MeA) and cortical (CoA) amygdala.

##### Statistical analysis

Descriptive statistics were evaluated using the psych package in R.^37^ Epilepsy onset age, disease duration, lifetime focal to bilateral tonic-clonic siezures (FBTCs), antiepileptic drug (AED) count and depressive symptoms were tested for significant differences between epilepsy subgroups, and multivariable linear models were evaluated for statistical significance using the *lm* function in base R (R Foundation, Vienna, Austria). Group differences in amygdala volume were tested by regressing normalized regional volume on group (no-HS, HS, control). Overall group effects for analyses involving multiple pairwise comparisons were assessed via omnibus *F*-tests using Anova in R. Adjusted *P*-values (*P*_adj_) for *a priori* pairwise contrasts (no-HS versus HS and no-HS versus control) were calculated using the Benjamini–Hochberg false discovery rate approach, which provides a balanced correction against both Type I and Type II error rates in relatively small, unbalanced clinical samples. Participants missing psychiatric measures were excluded from models including those variables and depressive symptoms were regressed on volume and its interaction with group. All multivariable models included confound variables for age and sex and extreme outliers were excluded from primary analysis based on Cook’s *D* > 1 if the observation was separated from the distribution.

To further evaluate the sensitivity and construct validity of the discretized transdiagnostic measure used in the Mechanistic cohort, we performed a secondary sensitivity analysis within the Discovery cohort. Continuous *T*-scores from the Depressive, Anxiety, and Attention Deficit Problems subscales were converted into dichotomized markers based on the recommended clinical cutpoint (*T* ≥ 65) and summed to create a 0–3 composite, mirroring both the discrete nature and the cumulative psychiatric burden of the data collected for the Mechanistic cohort.

### Mechanistic pilot cohort

#### Participants and recruitment

Patients with pharmacoresistant focal epilepsy and controls were recruited from UW Health outpatient clinics and the inpatient Epilepsy Monitoring Unit (EMU). Eligible participants were 18–55 years old. Patients demonstrated no gross abnormalities other than HS on prior clinical MRI and received a diagnosis of focal epilepsy supported by previously confirmed focal seizures via EMU EEG or at least two of the following: (i) history of seizures with semiology consistent with neurologist-determined focal epilepsy, (ii) EEG showing focal epileptiform abnormalities, (iii) MRI evidence of HS. Participants gave written informed consent prior to enrolment. Demographics and clinical variables were obtained from chart review, a seizure log and clinical interviews during an initial study visit at the University of Wisconsin-Madison. [^18^F]FEPPA PET/MR imaging, an *in vivo* index of regional gliosis,^38^ and blood samples were also obtained. Eligible participants gave written informed consent prior to the initial study visit. All procedures were approved by the Institutional Review Board of the University of Wisconsin School of Medicine and Public Health (Protocol #2017-0458). Patients were subsequently included in this study if they met the following criteria: available T1w MRI and [^18^F]FEPPA PET, unilateral seizure onset zone and available data for presence of HS.

#### Procedures

##### Psychiatric measurement

To assess psychiatric burden in the Mechanistic cohort and enable exploratory analysis of the interaction between gliosis and volume, a composite measure was derived from historical psychiatric problems documented in patient medical records. Medical charts were reviewed for historical documentation of depressive, anxiety or attention deficit problems—identified in the Discovery cohort as the most prevalent and representative symptom domains (Supplementary Fig. 2). Notably, this transdiagnostic grouping is supported by recent large-scale genomic analyses identifying a shared genetic liability across these problem domains.^39^ To capture the cumulative clinical impact, psychiatric burden was operationalized as a composite score ([0–3]), reflecting the count of documented conditions. This approach provided a pragmatic and ecologically valid transdiagnostic index of psychiatric burden suitable for this exploratory analysis. Psychiatric data were unavailable for controls from the Mechanistic cohort.

##### Anatomical and [^18^F]FEPPA PET/MR processing

All imaging was acquired on a GE Healthcare (Waukesha, WI) SIGNA simultaneous 3T PET/MR system using a dedicated 8-channel high-resolution head coil. High-resolution anatomical images used a 3D BRAVO sequence (TR/TE/TI: 8.5/3.248/450 ms; flip angle: 12°; FOV: 24 cm; acquisition matrix: 512 × 512; slice thickness: 1.2 mm). Anatomical images were processed using FreeSurfer (version 7.4.1) consistent with the Discovery cohort. [^18^F]FEPPA radiotracer was manufactured at the University of Wisconsin-Madison Waisman Center. [^18^F]FEPPA (mean injected dose = 283.75 ± 33.06 MBq) was administered via an indwelling catheter placed in the antecubital vein and PET data were acquired in dynamic mode for 90 min post-injection. Static PET images were motion-corrected and reconstructed between 50 and 70 min post-injection using a deep learning method with attenuation correction on par with CT scans.^40^ PETSurfer was used to coregister PET to anatomical parcellations. Consistent with volumetric measures, patient data were aligned to a common hemisphere (ipsilateral versus contralateral to the side of the seizure onset zone) and PET data were extracted from regions ipsilateral to side of seizure onset zone. Bilateral mean was calculated for controls. Given the limitations of PET resolution, FEPPA uptake was extracted from whole amygdala as an estimate of gliosis across subnuclei.

Partial volume correction (PVC) was implemented using the volume-based symmetric geometric transfer matrix method, which recovers mean regional activity, has been demonstrated superior to voxel-based methods for region-based analysis (particularly where age-related changes may be of concern^41^) and offers improved precision, noise propagation and robustness for smaller ROIs compared to the geometric transfer matrix method.^42^ To avoid dependency on a universally unaffected reference region (a particular challenge in a relatively diverse sample of multifocal epilepsy patients), FEPPA binding was quantified with standard uptake values (SUVs), which scale radiotracer uptake by injected dose and body weight. Sensitivity analyses were conducted to ensure robustness of findings regarding PVC and SUV quantification (see ‘Statistical analysis’ section).

##### Statistical analysis

Participants from the Mechanistic cohort were classified as high- (HAB) or mixed- (MAB) affinity based on translocator protein (*TSPO*) rs6971 genetic polymorphism to correct for known genetic variation in [^18^F]FEPPA binding affinity. Genotypes for participants missing serum data were imputed as previously described.^43^

Descriptive statistics and regression models were evaluated using R with procedures identical to the Discovery cohort. Clinical characteristics were further tested for significant differences between cohorts. The primary analysis testing the interactive effect of group and gliosis on volume was evaluated by regressing normalized regional volume on the interaction between group (no-HS, HS, control) and SUV. To explore the possible moderating effect of gliosis on the relationship between volume and psychiatric burden in no-HS, psychiatric burden was regressed on volume and its interaction with SUV.

FEPPA models included confound variables for age and sex and an additional nuisance covariate for binding affinity (HAB or MAB). For the primary Mechanistic analysis, outliers were treated identically to the Discovery cohort. Outliers were not excluded from the exploratory psychiatric analysis to preserve degrees of freedom. To evaluate robustness of PET measures with regard to SUV quantification, sensitivity analyses were conducted for significant results using standardized uptake value ratios (SUVRs) with a cerebellum pseudo-reference region, a common alternative to SUVs when volume of distribution measurements using plasma input functions are unavailable.^44^ Additionally, to ensure that relationships between volume and gliosis were not driven by PVC, sensitivity analyses were conducted using uncorrected PET data. The data from this study are available from the corresponding author, upon reasonable request and IRB approval.

### *Post hoc* analysis

To investigate the disease-related temporal dynamics of amygdala alterations, a *post hoc* linear regression was performed within the Discovery cohort patient group, regressing CeA volume on disease duration and its interaction with group (no-HS versus HS, controlling for age and sex). Additionally, descriptive analysis of a longitudinal case was conducted using difference scores (postictal minus interictal) to examine peri-ictal subnuclear volume changes for one patient from the Mechanistic cohort who underwent an interictal (2 weeks seizure free) and postictal [^18^F]FEPPA PET/MR scan after an EMU stay.

## Results

### Discovery cohort

#### Cohort characteristics

Participants in the Discovery cohort consisted of 43 patients with TLE (age = 42 ± 11 years, 23 female) and 80 controls (age = 35 ± 11 years, 42 female). From the 80 control participants, 17 did not have available psychiatric measures and were excluded from those analyses, leaving 63 controls (age = 36 ± 11 years, 31 female) and 43 patients. Patients reported significantly more depressive symptoms relative to controls (*b* = 3.29, *t*(102) = 3.52, *P* < 0.001, 95% CI [1.44, 5.14]).

Within the patient group from this cohort, 23 (age = 43 ± 10 years, 14 female) exhibited HS and 20 (age = 41 ± 13 years, 9 female) exhibited no-HS. Compared to HS, the no-HS group exhibited relatively later epilepsy onset, shorter disease duration, more lifetime FBTCs and fewer mean depressive symptoms (Table 1); However, none of these clinical differences reached statistical significance and only disease duration reached trend-level difference, such that duration was shorter in the no-HS group (*b* = −8.45, *t*(41) = −1.97, *P* = 0.056, 95% CI [−17.20, 0.23]). Demographic and clinical characteristics of all subgroups are presented in Table 1.

**Table 1:** Demographics and clinical characteristics by cohort

Measure	Subgroup	D [*M* ± SD]	M [*M* ± SD]
*N*	No-HS	20	8
	HS	23	2
	Control	80	9
Age (years)	No-HS	[41.05 ± 13]	[39.12 ± 13.17]
	HS	[42.83 ± 9.69]	[46.5 ± 16.26]
	Control	[35.34 ± 11]	[35.22 ± 10.2]
Sex (male/female)	No-HS	11/9	5/3
	HS	9/14	2/0
	Control	38/42	5/4
Onset Zone (left/right)	No-HS	12/8	4/4
	HS	20/3	1/1
Onset age (years)	No-HS	[26.32 ± 14.91]	[29.75 ± 11.32]
	HS	[19.61 ± 13.92]	[42.5 ± 17.68]
Duration (years)	No-HS	[14.72 ± 14.04]	[9.38 ± 8.73]
	HS	[23.21 ± 14.18]	[4 ± 1.41]
Lifetime FBTCs	No-HS	[17.73 ± 29.95]	[8.5 ± 16.79]
	HS	[7.33 ± 8.99]	[5.5 ± 7.78]
AED count	No-HS	[2.1 ± 0.91]	[1.75 ± 0.46]
	HS	[2 ± 1]	[3 ± 1.41]
Depressive Problems	No-HS	[7.05 ± 4.82]	–
	HS	[9.30 ± 5.41]	–
	Control	[4.79 ± 4.07]	–
Psychiatric burden	No-HS	–	[1.12 ± 0.83]
	HS	–	[1.5 ± 0.71]
	Control	–	–

Columns indicated D and M are Discovery and Mechanistic cohorts, respectively. Mean and standard deviation of continuous clinical characteristics are denoted in brackets. Two Discovery cohort patients with HS were missing FBTC data.

#### Amygdala is enlarged in patients with no-HS

##### Whole amygdala

*A priori* pairwise contrasts revealed enlarged whole ipsilateral amygdala volume in patients with no-HS compared to patients with HS (*b* = 1.65 × 10^−2^, *t*(118) = 3.73, *P* < 0.001, *P*_adj_ < 0.001, 95% CI [0.01, 0.03]) and controls (*b* = 1.11 × 10^−2^, *t*(118) = 3.04, *P* = 0.003, *P*_adj_ = 0.003, 95% CI [0.00, 0.02]; Table 2; Fig. 1A). The overall effect of group on whole ipsilateral amygdala volume was also significant (*F*(2,118) = 7.33, *P* = 0.001).

**Figure 1:**
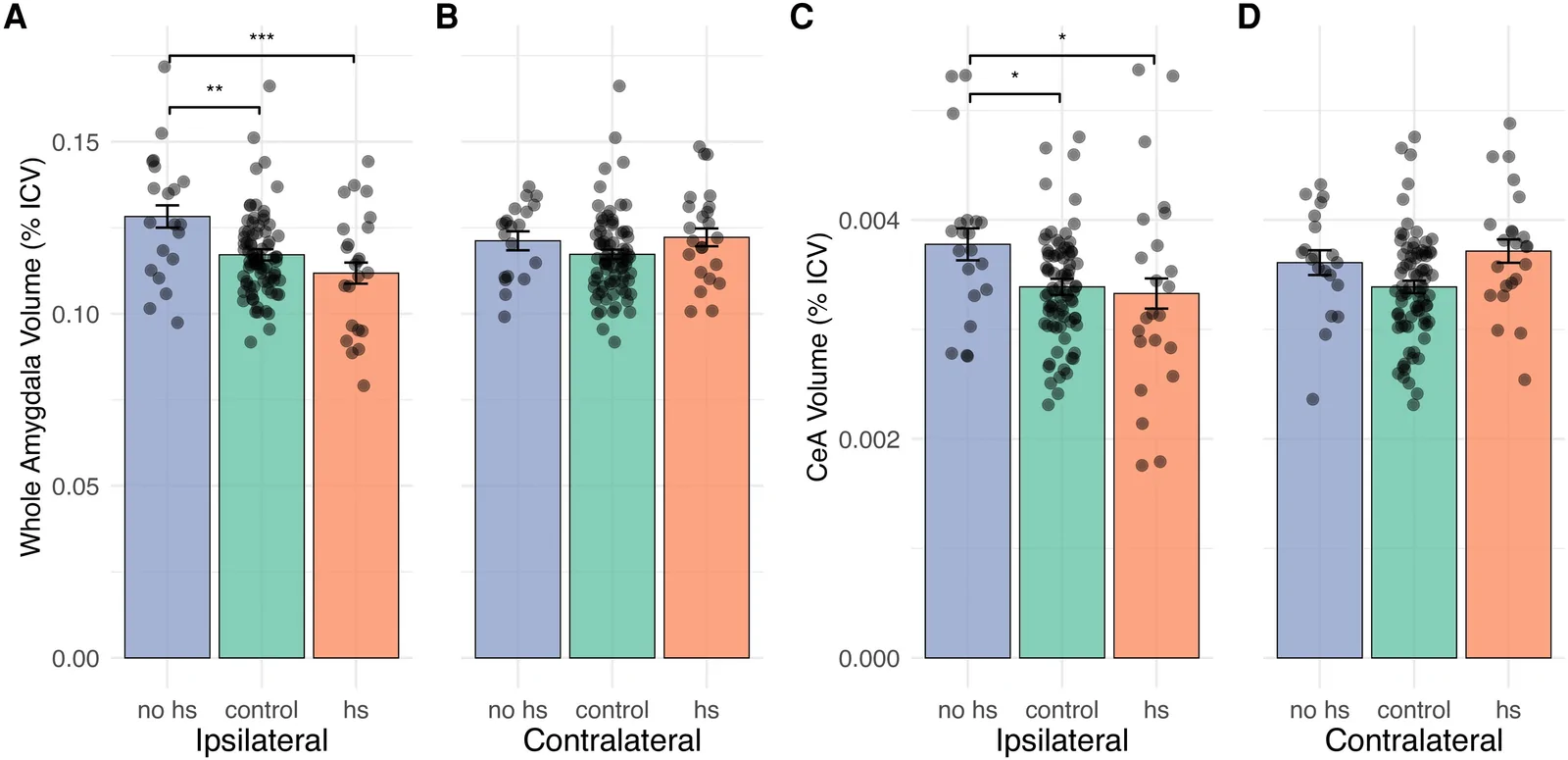
**Ipsilateral amygdala enlargement characterizes the no-HS phenotype.** (**A, B**) *A priori* pairwise contrasts (calculated using a multivariable linear model adjusting for age and sex) reveal that whole ipsilateral amygdala volume (% ICV) is significantly enlarged in patients without HS (*N* = 20) relative to both patients with HS (*t*(118) = 3.73, *P* < 0.001, *P*_adj_ < 0.001; *N* = 23) and controls (*t*(118) = 3.04, *P* = 0.003, *P*_adj_ = 0.003; *N* = 80) in the Discovery cohort, consistent with previous reports of ipsilateral AE in no-HS. (**C, D**) Enlargement of the ipsilateral CeA is consistent with the whole amygdala (no-HS versus HS: *t*(118) = 2.26, *P* = 0.026, *P*_adj_ = 0.026; no-HS versus control: *t*(118) = 2.36, *P* = 0.020, *P*_adj_ = 0.026). Raw data points and standard error bars are plotted over group means. Each data point represents an independent measure from a single participant. Markers indicate significant no-HS group differences (*P_adj_ < 0.05, **P_adj_ < 0.01, ***P_adj_ < 0.001).

**Table 2:** Results

Model	Dataset	Nucleus	Term	Statistic	Beta	*P*-value	*P*_adj_
Vol ∼ Group	D	Whole	No-HS > Ctrl	*t*(118) = −3.04	−1.113 × 10^–2^	0.0029	0.0029
			No-HS > HS	*t*(118) = −3.73	−1.646 × 10^–2^	0.0003	0.0006
	D	BLA	No-HS > Ctrl	*t*(118) = −3.17	−9.068 × 10^–3^	0.0019	0.0019
			No-HS > HS	*t*(118) = −3.72	−1.281 × 10^–2^	0.0003	0.0006
	D	CeA	No-HS > Ctrl	*t*(118) = −2.36	−3.885 × 10^–4^	0.0201	0.0256
			No-HS > HS	*t*(118) = −2.26	−4.494 × 10^–4^	0.0256	0.0256
	D	MeA	No HS > Ctrl	–	–	ns	ns
			No-HS > HS	*t*(118) = −1.67	−2.716 ×10^–4^	0.0973	ns
	D	CoA	No-HS > Ctrl	*t*(118) = −1.69	−1.644 × 10^–4^	0.0942	0.0942
			No-HS > HS	*t*(118) = −2.66	−3.123 × 10^–4^	0.0089	0.0178
Dep ∼ Vol × Group	D	Whole	Vol × Ctrl	*t*(98) = 3.10	2.269 × 10^2^	0.0025	0.0051
			Vol × HS	*t*(98) = 2.17	1.658 × 10^2^	0.0323	0.0323
	D	BLA	Vol × Ctrl	*t*(98) = 3.08	2.853 × 10^2^	0.0027	0.0054
			Vol × HS	*t*(98) = 2.12	2.087 ×10^2^	0.0368	0.0368
	D	CeA	Vol × Ctrl	*t*(98) = 2.24	4.194 × 10^3^	0.0272	0.0406
			Vol × HS	*t*(98) = 2.08	3.551 × 10^3^	0.0406	0.0406
	D	MeA	Vol × Ctrl	–	–	ns	ns
			Vol × HS	–	–	ns	ns
	D	CoA	Vol × Ctrl	*t*(98) = 2.45	7.302 × 10^3^	0.0159	0.0319
			Vol × HS	*t*(98) = 1.85	5.429 × 10^3^	0.0678	0.0678
Vol ∼ Group × SUV	M	Whole	SUV × Ctrl	*t*(9) = 2.62	0.05	0.0279^a^	0.0558
			SUV × HS	–	–	ns	ns
	M	BLA	SUV × Ctrl	*t*(9) = 2.51	0.04	0.0334^a^	0.0667
			SUV × HS	–	–	ns	ns
	M	CeA	SUV × Ctrl	*t*(9) = 3.33	1.719 × 10^–3^	0.0088^a,b^	0.0176^b^
			SUV × HS	–	–	ns	ns
	M	MeA	SUV × Ctrl	–	–	ns	ns
			SUV × HS	–	–	ns	ns
	M	CoA	SUV × Ctrl	*t*(9) = 2.47	1.016 × 10^–3^	0.0358	0.0716
			SUV × HS	–	–	ns	ns
Burden ∼ Vol × SUV	M	Whole	Vol × SUV	–	–	ns	–
	M	BLA	Vol × SUV	–	–	ns	–
	M	CeA	Vol × SUV	*t*(1) = 36.91	1.810 × 10^3^	0.0173^a^	–
	M	MeA	Vol × SUV	*t*(1) = −7.84	−1.232 × 10^4^	0.0807	–
	M	CoA	Vol × SUV	–	–	ns	–

Results presented are all from the ipsilateral side. ns indicates *P* > 0.1 for specific parameter in the given model. Dataset D and M are Discovery and Mechanistic cohorts, respectively. Volume (Vol); standardized uptake values (SUVs); basolateral (BLA); central (CeA); medial (MeA); cortical (CoA); control participant (Ctrl); hippocampal sclerosis (HS).

^a^Sensitivity model with SUVR was significant.

^b^Sensitivity model with PET uncorrected for PVE was significant.

##### Subnuclear specificity

The most consistent subnuclear effects were identified in the CeA and BLA. In the Discovery cohort, only CeA and BLA were enlarged in no-HS relative to both HS (CeA: *b* = 4.49 × 10^−4^, *t*(118) = 2.26, *P* = 0.026, *P*_adj_ = 0.026, 95% CI [5.59 × 10^−5^, 8.43 × 10^−4^]; BLA: *b* = 0.01, *t*(118) = 3.72, *P* < 0.001, *P*_adj_ < 0.001, 95% CI [0.01, 0.02]) and controls (CeA: *b* = 3.89 × 10^−4^, *t*(118) = 2.36, *P* = 0.020, *P*_adj_ = 0.026, 95% CI [6.19 × 10^−5^, 7.15 × 10^−4^]; Fig. 1C; BLA: *b* = 0.01, *t*(118) = 3.17, *P* = 0.002, *P*_adj_ = 0.002, 95% CI [0.00, 0.01]). No-HS MeA and CoA were not significantly different from controls, although no-HS CoA volume was significantly greater than HS (*b* = 3.12 × 10^−4^, *t*(118) = 2.66, *P* = 0.009, *P*_adj_ = 0.018, 95% CI [7.99 × 10^−5^, 5.45 × 10^−4^]). The overall effect of group on volume was also significant for CeA (*F*(2,118) = 3.26, *P* = 0.042), BLA (*F*(2,118) = 7.46, *P* < 0.001) and CoA (*F*(2,118) = 3.54, *P* = 0.032).

#### Amygdala volume is associated with depressive symptoms in patients with no-HS

##### Whole amygdala

Reduced whole ipsilateral amygdala volume within no-HS was associated with higher depressive symptoms relative to both HS (*b* = 165.77, *t*(98) = 2.17, *P* = 0.032, *P*_adj_ = 0.032, 95% CI [14.32, 317.21]) and controls (*b* = 226.11, *t*(98) = 3.10, *P* = 0.003, *P*_adj_ = 0.005, 95% CI [81.60, 372.21]). The overall group by volume interaction effect was also significant (*F*(2,98) = 4.97, *P* = 0.009). No association between volume and depressive symptoms was found in patients with HS or controls.

##### Subnuclear specificity

Again only reduced central and basolateral subnuclear volumes within no-HS of the Discovery cohort were associated with higher depressive symptoms relative to both patients with HS (CeA: *b* = 3.55 × 10^3^, *t*(98) = 2.08, *P* = 0.041, *P*_adj_ = 0.041, 95% CI [1.55 × 10^2^, 6.95 × 10^3^]; BLA: *b* = 209, *t*(98) = 2.12, *P* = 0.037, *P*_adj_ = 0.037, 95% CI [13.07, 404.39]) and controls (CeA: *b* = 4.19 × 10^3^, *t*(98) = 2.24, *P* = 0.027, *P*_adj_ = 0.041, 95% CI [4.83 × 10^2^, 7.90 × 10^3^]; Fig. 2A; BLA: *b* = 2.85, *t*(98) = 3.08, *P* = 0.003, *P*_adj_ = 0.005, 95% CI [101.21, 469.30]). Neither MeA nor CoA volume were differentially associated with depressive symptoms in no-HS relative to HS and of the two only reduced CoA volume within no-HS was associated with higher depressive symptoms relative to controls (*b* = 7.30 × 10^3^, *t*(98) = 2.45, *P* = 0.016, *P*_adj_ = 0.032, 95% CI [1.39 × 10^3^, 1.32 × 10^4^]). The overall group by volume interaction effect was significant for the BLA (*F*(2,98) = 4.89, *P* = 0.009) and CoA (*F*(2,98) = 3.13, *P* = 0.048).

**Figure 2:**
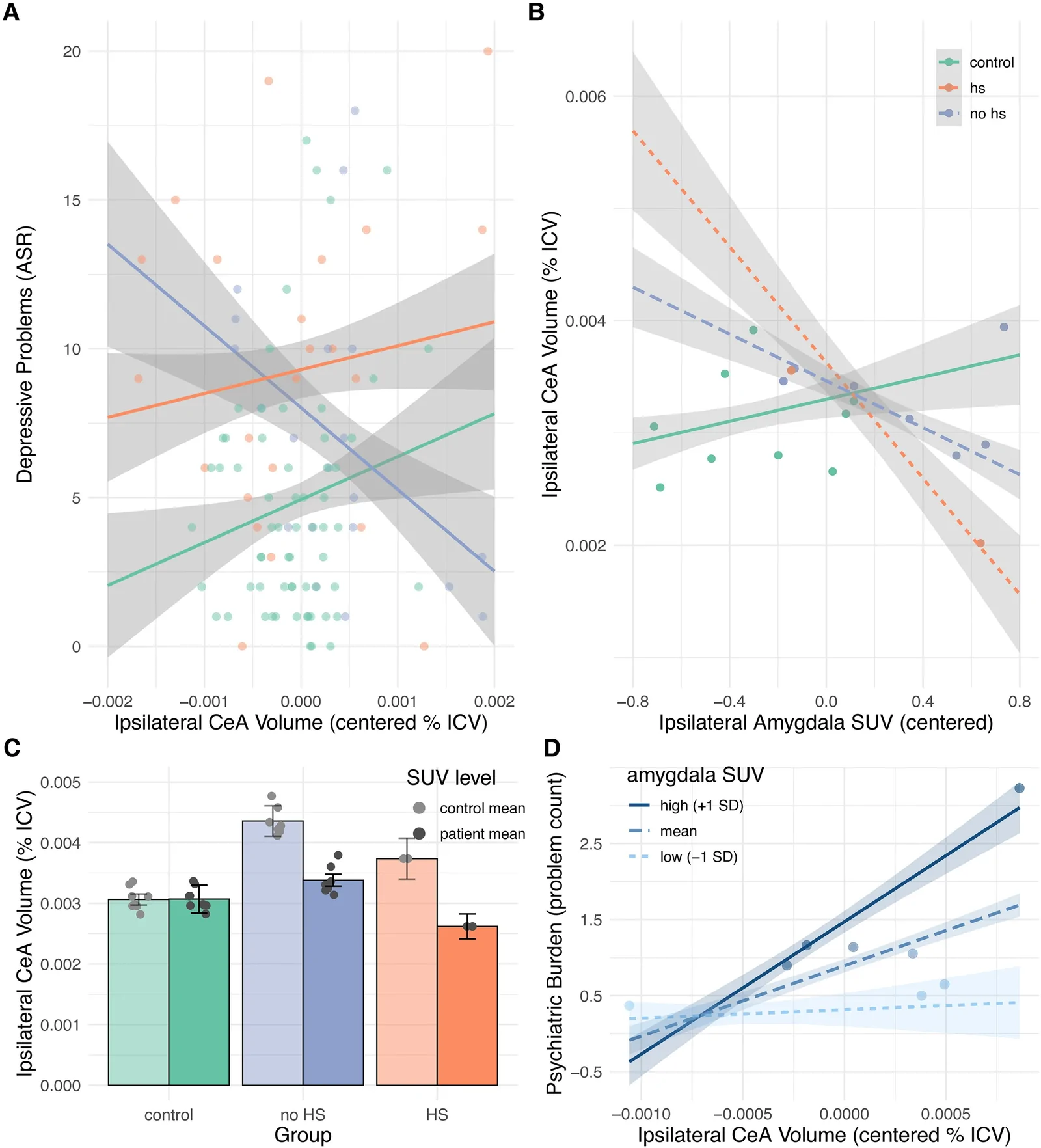
**Structural alterations are associated with gliosis and psychiatric burden.** (**A**) Reduced CeA volume is associated with increased ASR Depressive Problems in the no-HS group (*N* = 20) of the Discovery cohort relative to both HS (*t*(98) = 2.08, *P* = 0.041, *P*_adj_ = 0.041; *N* = 23) and controls (*t*(98) = 2.24, *P* = 0.027, *P*_adj_ = 0.041; *N* = 63) adjusted for age and sex. (**B**) Higher [^18^F]FEPPA SUV is significantly associated with reduced CeA volume in the no-HS group relative to controls adjusted for age, sex and PET binding affinity (*t*(9) = −3.33, *P* = 0.009, *P*_adj_ = 0.018; *N* = 7 no-HS, 2 HS, 9 controls), suggesting gliosis-related atrophy. (**A, B**) Multivariable linear model results are displayed with standard error bands and raw or mean-centred (*x*-axis) data points. Individual data points represent independent measures from a single participant. (**C**) Adjusting for the negative effect of gliosis on volume in the Mechanistic cohort reveals a predicted amygdala volume consistent with the canonical AE phenotype. Individual bars represent estimated marginal means extracted from the multivariable model in **B** for each group. Lighter bars depict estimated marginal means adjusted to baseline SUV (control mean), illustrating the significant CeA enlargement in the no-HS group (*N* = 7) relative to both HS (*t*(9) = 2.45, *P* = 0.037, *P*_adj_ = 0.037; *N* = 2) and controls (*t*(9) = 3.65, *P* = 0.005, *P*_adj_ = 0.011, *N* = 9) when the atrophic effect of gliosis is statistically removed. Darker bars depict estimated marginal means at mean patient SUV levels, reflecting the atrophic effect of gliosis in patients. Individual data points represent observations from a single participant adjusted for covariates at the corresponding group mean SUV. (**D**) Exploratory partial residual interaction plot illustrates a possible moderating effect of gliosis on CeA volume and psychiatric burden in patients with no-HS (*t*(1) = 36.91, *P* = 0.017, adjusted for age, sex and PET binding affinity; *N* = 8). Multivariable linear model results are displayed with 95% confidence intervals and individual residualized data points representing independent measures from a single patient. The association between volume and psychiatric burden appears to be driven by patients with elevated gliosis, consistent with an active neuroimmune state driving both volume alterations and symptoms.

#### Contralateral control analysis

On the contralateral side, only no-HS CoA volume was greater than controls (*b* = 1.77 × 10^−4^, *t*(118) = 2.11, *P* = 0.037, *P*_adj_ = 0.074, 95% CI [1.11 × 10^−5^, 3.43 × 10^−4^]) in the Discovery cohort. The overall effect of group on contralateral CoA volume was not significant (*F*(2,118) = 2.23, *P* = 0.112).

#### Outlier and sensitivity analysis

No model outliers were identified in the Discovery cohort. The sensitivity analysis using the surrogate 0–3 composite psychiatric measure revealed a significant ‘group’ by ‘volume’ interaction such that reduced CeA volume in no-HS was associated with greater psychiatric burden relative to both patients with HS (*b* = −8.40 × 10^2^, *t*(98) = −2.73, *P* = 0.007, *P*_adj_ = 0.010, 95% CI [−1449.48, −229.80]; Supplementary Fig. 3) and controls (*b* = −8.79 × 10^2^, *t*(98) = −2.62, *P* = 0.010, *P*_adj_ = 0.010, 95% CI [−1545.60, −212.70]), reproducing the effect observed in the primary analysis of depressive symptoms (Fig. 2A). The overall group by volume interaction effect was also significant (*F*(2,98) = 4.47, *P* = 0.014).

### Mechanistic pilot cohort

#### Cohort characteristics

The Mechanistic cohort consisted of 10 patients (age = 41 ± 13 years, 3 females) with pharmacoresistant focal epilepsy (*n* = 5 temporal, *n* = 3 temporal plus and *n* = 2 frontal) and 9 controls (age = 35 ± 10 years, 4 females). Genotype for one participant missing serum data was imputed, resulting in 6 HAB (4 MAB) patients and 5 HAB (4 MAB) controls.

Within the patient group, eight patients (age = 39 ± 13 years, 3 female) exhibited no-HS and two patients (age = 47 ± 16 years, 0 female) exhibited HS (Table 1). In this cohort, patients with no-HS were prescribed fewer AEDs than the two HS patients (*b* = −1.25, *t*(8) = −2.39, *P* = 0.044, 95% CI [−2.46, −0.04]). Furthermore, the two patients with HS in the Mechanistic cohort exhibited a significantly later age of epilepsy onset (mean = 42.5 years) compared to the HS subgroup in the Discovery cohort (*b* = 22.89, *t*(23) = 2.20, *P* = 0.038, 95% CI [1.37, 44.40]). There were no other significant differences when comparing clinical characteristics across Mechanistic subgroups or across cohorts.

#### Amygdala volume alterations are associated with gliosis

##### Whole amygdala

The ‘group’ by ‘SUV’ model revealed a significant interaction effect such that elevated SUV was associated with trend-level reductions in whole amygdala volume in no-HS relative to controls (no-HS: *b* = −0.05, *t*(9) = −2.62, *P* = 0.028, *P*_adj_ = 0.056, 95% CI [−0.10, −0.01]; Table 2), suggesting that macroscopic volumetric heterogeneity in no-HS may reflect a balance between pathological tissue hypertrophy and neuroinflammation-related tissue loss. The overall group by SUV interaction effect reached trend level (*F*(2,9) = 4.07, *P* = 0.055).

##### Subnuclear specificity

The ‘group’ by ‘SUV’ interaction effect was significant such that greater SUV was associated with larger reductions in CeA (*b* = −1.72 × 10^−3^, *t*(9) = −3.33, *P* = 0.009, *P*_adj_ = 0.018, 95% CI [−2.89 × 10^−3^, −5.51 × 10^−4^]), and trend level for BLA (*b* = −0.04, *t*(9) = −2.51, *P* = 0.033, *P*_adj_ = 0.067, 95% CI [−0.08, −0.00]), and CoA (*b* = −1.02x10^−3^, *t*(9) = −2.47, *P* = 0.036, *P*_adj_ = 0.072, 95% CI [−1.95 × 10^−3^, −8.39 × 10^−5^]) volume in no-HS relative to controls. The overall group by SUV interaction effect was also significant for CeA (*F*(2,9) = 6.37, *P* = 0.019) and trend level for BLA (*F*(2,9) = 3.87, *P* = 0.061) and CoA (*F*(2,9) = 3.077, *P* = 0.096). Differences in simple slopes illustrate how the relationship between SUV and volume differs between control and patient groups (Fig. 2B). Pairwise group differences in volume at mean-centred SUV revealed greater volume only in the CeA relative to both HS (*b* = 6.92 × 10^−4^, *t*(9) = 2.45, *P* = 0.037, *P*_adj_ = 0.037, 95% CI [5.27 × 10^−5^, 1.33 × 10^−3^]) and controls (*b* = 8.03 × 10^−4^, *t*(9) = 3.65, *P* = 0.005, *P*_adj_ = 0.011, 95% CI [3.06 × 10^−4^, 1.30 × 10^−3^]) and a significant overall effect of group (*F*(2,9) = 7.17, *P* = 0.014). Estimated marginal means extracted from this model illustrate the predicted CeA volume for each group at the control and patient mean level of SUV (Fig. 2C). MeA was significantly larger in no-HS relative only to HS (*b* = 1.01 × 10^−3^, *t*(9) = 2.69, *P* = 0.025, *P*_adj_ = 0.049, 95% CI [1.60 × 10^−4^, 1.85 × 10^−3^]), but the overall effect of group on MeA volume was not significant (*F*(2,9) = 3.69, *P* = 0.067).

#### Moderating effect of gliosis on amygdala volume and psychiatric burden

Exploratory analysis testing whether [^18^F]FEPPA SUV moderates the link between volume and psychiatric burden in patients with no-HS revealed a significant positive interaction effect localized to the CeA (*b* = 1.81 × 10^3^, *t*(1) = 36.91, *P* = 0.017, 95% CI [1187.12, 2433.72]; Fig. 2D).

#### Contralateral control analysis

In the psychiatric analysis, greater contralateral SUV interacted with CeA volume such that the association between volume and burden was less positive in individuals with greater SUV (*b* = −7.22 × 10^3^, *t*(1) = −23.53, *P* = 0.027, 95% CI [−1.11 × 10^4^, −3.32 × 10^3^]); in contrast to ipsilateral results, this contralateral interaction lost significance in the SUVR sensitivity analysis. No other significant differences were found on the contralateral side.

#### Outlier and sensitivity analysis

One patient was consistently identified as an influential outlier in the Mechanistic cohort. Notably, this individual’s PET scan was characterized by particularly low whole-brain [^18^F]FEPPA uptake (nearly 1.5 SDs below the control mean). Importantly, this patient was not identified as an influential outlier in SUVR sensitivity models, suggesting that their influence was driven by between-subject differences in whole-brain [^18^F]FEPPA uptake, which may be more effectively mitigated by normalization to a reference region. Accordingly, SUVR sensitivity analyses were generally consistent with SUV results. ‘Group’ by ‘SUVR’ interaction effects maintained unadjusted *P* < 0.05 for the whole amygdala, BLA and CeA, whereas only the CoA model dropped below threshold. The moderating effect of gliosis on CeA volume and psychiatric burden remained significant. Finally, sensitivity analyses using uncorrected PET data confirmed an inverse relationship between volume and gliosis in no-HS relative to controls across all amygdala ROIs. Notably, the interactive effect on CeA volume remained statistically significant. While our exploratory psychiatric model in the Mechanistic cohort lost significance in this uncorrected PET sensitivity analysis, the direction of this effect also remained consistent.

### *Post hoc* analysis: disease progression and state dependence

To explore the potential effect of disease-related dynamics on the observed amygdala volume alterations, we conducted additional *post hoc* analysis to explore the temporal and state-dependent nature of these changes.

#### Association with disease duration (Discovery cohort)

Within the Discovery cohort, we tested whether CeA volume was associated with disease duration (controlling for age and sex). Results revealed a significant reduction in volume specifically in patients with no-HS as disease duration increases (*b* = −3.73 × 10^−5^, *t*(37) = −2.66, *P* = 0.012, 95% CI [−6.57 × 10^−5^, −8.88 × 10^−6^]; Fig. 3B), suggesting that the observed CeA enlargement may be most prominent in earlier stages of the disease and diminish as the disease progresses. In contrast, no significant association between CeA volume and disease duration was observed in patients with HS (*P* > 0.05). The interaction term was not significant.

**Figure 3:**
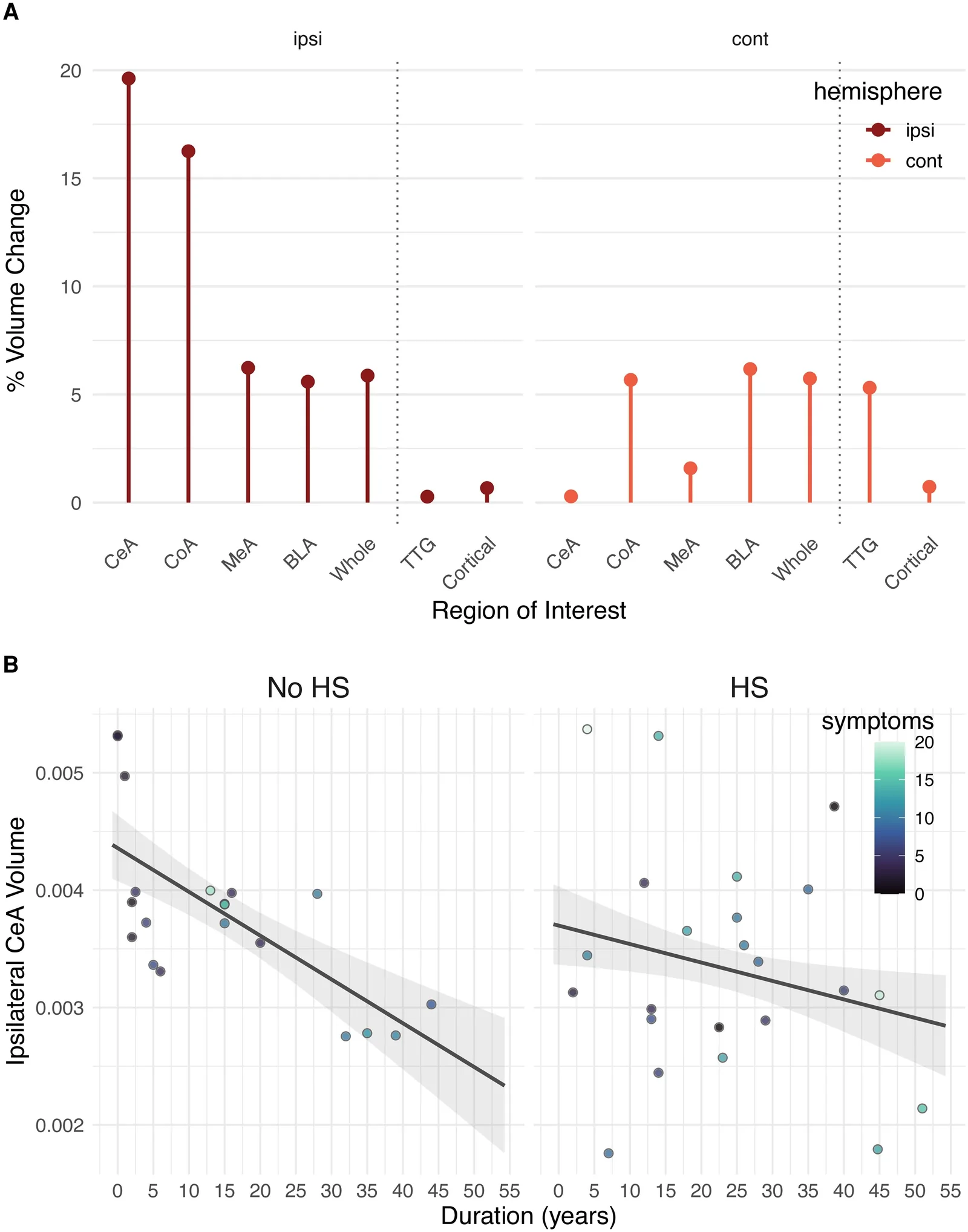
**Disease-related amygdala volume dynamics reflect a state-dependent and progressive pathology.** (**A**) Longitudinal peri-ictal descriptive case analysis of a single subject from the Mechanistic cohort reveals transient ipsilateral (ipsi) enlargement of the central amygdala (CeA) and cortical amygdala (CoA) in the postictal state compared to baseline. This volumetric change was not observed in the similarly sized medial amygdala (MeA), the basolateral amygdala (BLA), in control regions (i.e. transverse temporal gyrus, TTG; cortical mean) or on the contralateral (cont) side, highlighting the potentially unique susceptibility of the CeA to acute seizure activity. (**B**) In the no-HS phenotype, longer disease duration is associated with a significant reduction in CeA volume (*t*(37) = −2.66, *P* = 0.012, controlling for age and sex; *N* = 20 no-HS, 23 HS), suggesting progressive atrophy of the initially enlarged structure over time. Multivariable linear model results are displayed with raw data points and standard error bands overlaid. Each data point represents independent measures from a single patient. Point colour represents depressive symptoms (Adult Self-Report Depressive Problems), illustrating that while greater depressive symptoms within no-HS are generally observed in the context of reduced volume, variation in depressive symptoms occur across the volumetric range. Notably, the acute enlargement (**A**) contrasts with the reduced volume observed over time (**B**), highlighting distinct structural responses to acute versus chronic disease-related effects.

#### Disease-state dependence (Mechanistic cohort)

We further explored whether acute disease states contribute to CeA volume alterations through descriptive analysis of a longitudinal peri-ictal case. Analysis revealed postictal enlargement localized to the CeA (19.6%) and CoA (16.3%; Fig. 3A), suggesting that transient changes may oppose chronic disease-related volume reductions.

## Discussion

Although AE and altered neuroimmune pathways are both implicated in no-HS epilepsy and psychiatric illness, the potential role of gliosis as a common substrate linking AE to psychiatric burden in epilepsy remains poorly understood. Findings presented here demonstrate ipsilateral amygdala enlargement and a robust association between reduced ipsilateral amygdala volume and elevated depressive symptoms in patients with no-HS focal epilepsy—an effect not seen in patients with HS or in typical controls. In an independent cohort, reduced volume in patients with no-HS is further linked to elevated *in vivo* gliosis, suggesting a process of gliosis-related atrophy despite an overall phenotype of enlargement. While gliosis is likely both a cause and consequence of seizure activity, our exploratory analysis provides supporting evidence that the AE phenotype common to both no-HS epilepsy and psychiatric illness may converge on an altered neuroimmune state.

### Gliosis as a mechanism

Amygdala enlargement is reported in both no-HS epilepsy and primary psychiatric research—two highly comorbid diseases. While recent evidence from a heterogeneous sample of patients with pharmacoresistant focal epilepsy demonstrated an association between AE and psychiatric symptoms,^20^ our data from a representative Discovery sample of patients with TLE show the opposite—that reduced volume is associated with more symptoms in no-HS relative to patients with HS or healthy controls (Fig. 2A).

These seemingly discordant findings are reconciled by PET findings from our Mechanistic dataset, where we identified an association between elevated gliosis and lower amygdala volume in patients without evidence of sclerotic lesions (Fig. 2B). This suggests that, whereas AE is often observed across patients with no-HS, gliosis can mask this phenotype on MRI in some individuals. We propose that, while AE may be a proximal marker for underlying pathological gliosis and psychiatric risk, chronic inflammation can drive progressive tissue loss, masking AE over time even when pathological gliosis and high psychiatric risk remain. Consequently, in a sample with broader range and longer average disease duration, such as our Discovery cohort, atrophy becomes the dominant effect observed on MRI, driving an inverse relationship between volume and symptoms.

Supporting this interpretation, our exploratory model of psychiatric burden illustrates the hypothesized association between greater volume and increased psychiatric burden in patients with no-HS—an effect localized to the central subnucleus which appears to be driven by individuals with a higher degree of gliosis (Fig. 2D). Together, these Mechanistic findings suggest that the divergence between our Discovery findings and prior literature may be attributable to clinical characteristics and methodological differences which highlight opposing effects of gliosis over time.

### Sample characteristics

Mourre and colleagues^20^ found a positive correlation between psychiatric symptoms and amygdala volumes only when AE was categorically identified based on *z*-score cut-offs and the low volume group was excluded. By excluding atrophic cases, the volumetric signal in their remaining sample may have been dominated by patients with elevated gliosis and little-to-no atrophy. Crucially, our findings illustrate that normal or reduced amygdala volume is ambiguous and captures the net result of competing processes: pathological hypertrophy and gliosis-related atrophy (Fig. 4).

**Figure 4:**
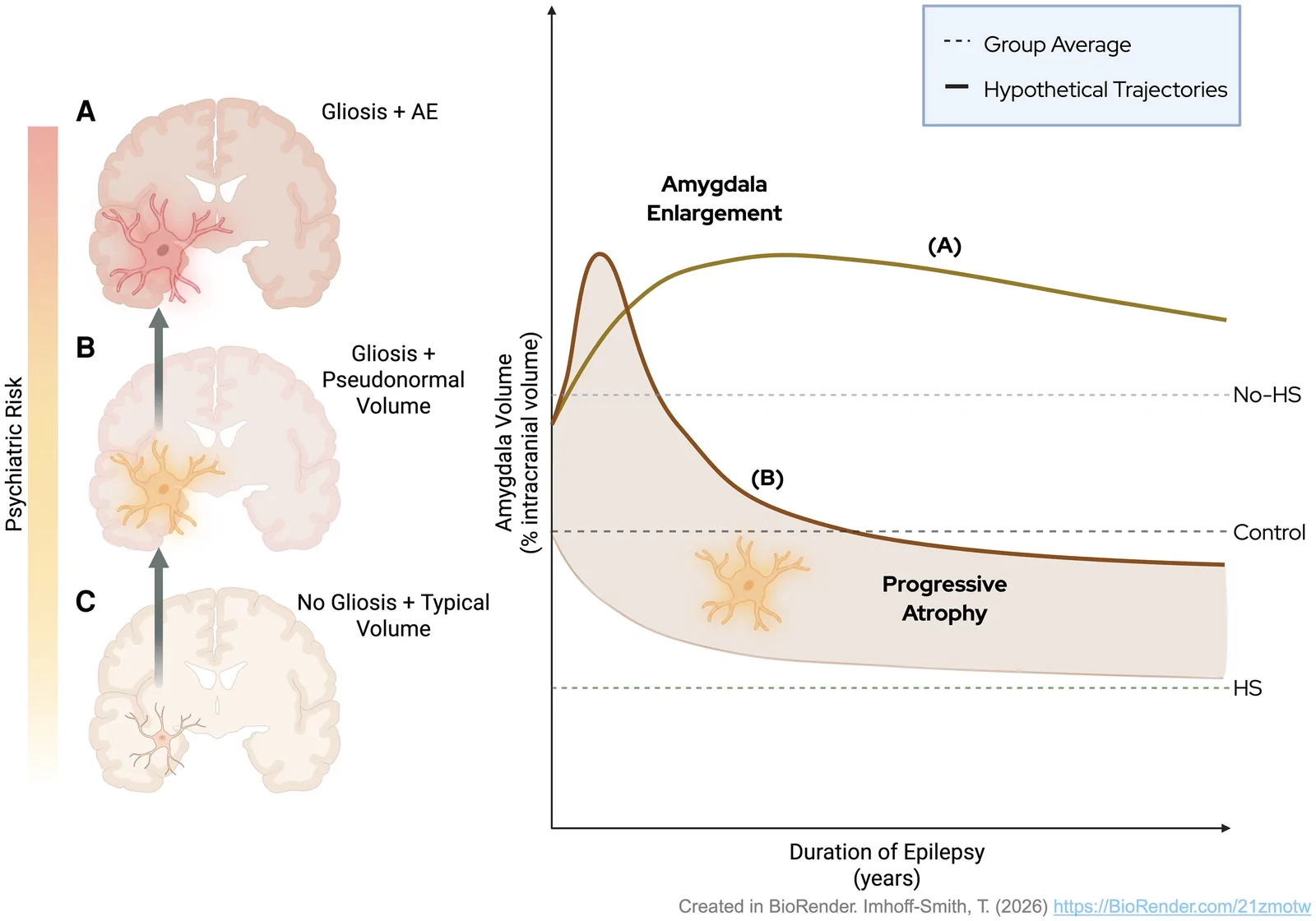
**Conceptual model of gliosis-related masking and psychiatric risk.** (**Left**) Hypothetical clinical phenotypes illustrate the relationship between gliosis, volume and psychiatric risk. (**Right**) Hypothetical disease trajectories (solid lines) illustrate that macroscopic amygdala volume captures the net result of two competing processes: pathological neuroinflammation (driving volume increase) and progressive atrophy (driving volume loss). The intersection of these trajectories with the Control group average demonstrates how a ‘normal’ volume reading can be ambiguous. Letters (**A–C**) correspond to hypothetical phenotypes observed at specific timepoints along these theoretical trajectories. (**A**) Gliosis + AE represents a state where neuroinflammation coincides with minimal tissue loss, resulting in overt macroscopic enlargement. This phenotype illustrates a high degree of gliosis-related enlargement and, consequently, the greatest psychiatric risk. (**B**) Gliosis + Pseudonormal Volume illustrates the masking effect. In this state, the hypertrophic effect of neuroinflammation is offset by concurrent tissue loss, and the net volume appears normal. Note that while the extent of structural atrophy obscures the macroscopic phenotype of AE, the underlying hypertrophic processes remain, conferring elevated risk for psychiatric illness. (**C**) No Gliosis + Typical Volume represents a homeostatic baseline with typical volume and reduced psychiatric risk. Created in BioRender. Imhoff-Smith, T. (2026). https://BioRender.com/21zmotw.

Through our Mechanistic cohort, which, like the Mourre sample, consisted mostly of patients undergoing ictal monitoring with challenging, pharmacoresistant disease, we quantified enlargement as a continuous variable and statistically isolated gliosis-related structural alterations using regional [^18^F]FEPPA PET. In this cohort, however, only once the effect of gliosis was statistically accounted for did the AE phenotype emerge (Fig. 2C). This is consistent with the idea that amygdala alterations in no-HS are driven by pathogenic neuroimmune processes that can lead to atrophy and suppress the control-relative enlargement in at least some patients. Our multi-modal analysis suggests that AE is not a static hypertrophy, but rather a dynamic state where active neuroimmune processes can introduce structural heterogeneity and obscure an overall phenotype of tissue enlargement.

In contrast, our Discovery sample from the Epilepsy Connectome Project was a largely outpatient clinic sample recruited to be representative of the general population of TLE and is inclusive of patients with longer duration and chronic epilepsy. Statistically, these no-HS patients exhibited the expected phenotype of AE; however, even within this group, *post hoc* analyses revealed a significant negative correlation between disease duration and amygdala volume (Fig. 3B). Notably, this progressive volume reduction was specific to the no-HS group and volume did not correlate with disease duration in patients with HS. In this general population sample of patients with no-HS, we speculate that persistent low-grade neuroinflammation drives initial enlargement followed by slow, progressive atrophy over time that results in the observed negative correlation between volume and depressive symptoms.

As reported by Grote *et al*., gliosis manifests differentially in a subset of patients with no-HS (I^2^GO);^7^ this distinct phenotype of reactive astrogliosis, characterized by glial hypertrophy in the relative absence of neuron loss and fibrillary scarring, could contribute to the macroscopic tissue enlargement quantified here and by others. While glia-associated hypertrophy possibly drives macroscopic amygdala enlargement in I^2^GO, our findings indicate that atrophy may also suppress this phenotype on clinical MRI in some patients without HS. Consequently, an in vivo measure of gliosis is necessary to disentangle the active neuroimmune state from the gross structural phenotype, potentially capturing individual psychiatric risks that are otherwise obscured by the opposing effects of hypertrophy and atrophy.

### Subnuclear specificity: the central amygdala

Our results identify the most robust effects in the ipsilateral CeA, consistent with a localized, disease-driven process rather than a whole-brain effect. The relative subnuclear specificity in our statistical models is recapitulated in our *N* = 1 case where examination of postictal volumetric changes revealed the greatest structural enlargement in CeA (19.6%), illustrating the possibly unique vulnerability of the central subnucleus to dynamic, disease-related plasticity.

Data linking psychiatric symptoms to subnuclear volumes in epilepsy are scarce. Notably, convergence of our findings across two cohorts associating CeA volume alterations with psychiatric burden aligns with the report by Mourre *et al*.,^20^ which found CeA-specific enlargement in patients with positive screening for depression and more severe anxiety. A second study by Ballerini and colleagues^4^ found larger ipsilateral lateral subnucleus in patients with a history of any psychiatric disorder; however, the Ballerini study used model covariates to adjust for total ICV, whereas the present study and Mourre normalized by total ICV for a within-subject control. This methodological difference may be responsible for the different findings.

Physiologically, the CeA is an autonomic hub characterized by dense coupling with the brainstem and with a high density of corticotropin-releasing factor neurons, positioning it as a critical mediator of anxiety, fear and systemic stress responses.^45,46^ The CeA may be uniquely vulnerable to inflammation; beyond its implication in immune-related psychiatric illness, preclinical evidence demonstrates its critical role in modulating the immune system in response to stress.^46-48^ Thus, pathogenic gliosis and hyperexcitability of these connections in no-HS epilepsy may lead to hormonal and physiological dysfunction and, consequently, disordered mood states.

### Multiple pathways to psychiatric illness

Critically, these results do not explain the high comorbidity in patients with HS. Despite similarly elevated psychiatric symptoms in patients with HS and no-HS, amygdala volume did not correlate with psychiatric symptoms in our Discovery HS group. This is consistent with the idea that there may be multiple pathways to psychiatric illness in patients with epilepsy.^49,50^ One pathway, driven by divergent patterns of neuroimmune activation and gliosis, may be an independent pathology that both drives a subset of epilepsy with the no-HS phenotype and predisposes those patients to an increased burden of psychiatric symptoms and immune-related psychiatric illness. In other patients with epilepsy, such as patients with HS, mood disorders may be elicited by seizure-driven cognitive and network dysfunction.^51^

### Limitations

While this study benefits from a design that utilizes the same methodology across two independent datasets, limitations exist. First, [^18^F]FEPPA PET is resource-intensive, resulting in a small Mechanistic dataset to test the interaction with gliosis. Furthermore, while substantially larger and more representative of the general TLE population than the Mechanistic cohort, the Discovery sample remains relatively modest, and the generalizability of these findings should be further evaluated in larger multicentre datasets. However, consistent findings on the ipsilateral side and consistency across sensitivity analyses and with previous literature support the robustness of these preliminary results.

Second, the estimate of inflammation was derived from the whole amygdala due to PET resolution constraints. Therefore, while our structural findings demonstrate subnuclear specificity, our PET data provide a regional index of gliosis rather than a spatially resolved map of subnuclear inflammation. It is possible that the neuroinflammatory signal is concentrated in the CeA, and partial volume effects cause this hotspot to elevate signal across the ROI. Alternatively, inflammation may be diffuse, with the CeA uniquely vulnerable to its effects. To address potential confounds due to signal spillover and recover mean amygdala activity, our primary analysis incorporated PVC. However, acknowledging that PVC might introduce artificial volume-related signal, we reran models using PET data uncorrected for partial volume effects. Critically, the inverse relationship between volume and gliosis in the no-HS group persisted, and significant structural correlations were restricted to the CeA, arguing against findings being a methodological artefact of PVC.

Finally, while psychiatric outcomes in the Discovery cohort were assessed using psychometrically validated measures of depressive symptoms from both patients and controls, the Mechanistic cohort relied on a lifetime transdiagnostic index obtained from the medical chart. Consequently, the exploratory Mechanistic analysis captures historical burden, whereas the Discovery analysis reflects current symptoms. Other limitations of chart review include reliance on heterogeneous diagnostic sources (PCP or psychiatrist), potential inconsistencies in documentation across providers and the unavailability of comparable control data. However, our sensitivity analysis using a discretized measure of psychiatric burden within the Discovery cohort reproduced our primary findings. This replication suggests that, while not temporally equivalent, the composite transdiagnostic psychiatric index is sufficiently sensitive to detect signals from the same underlying biobehavioural construct.

### Implications and future directions

Our findings provide preliminary *in vivo* evidence that amygdala volume alterations in a subset of patients with no-HS focal epilepsy are not purely structural, but reflect a state-dependent glial process associated with elevated psychiatric burden. We identify ipsilateral amygdala enlargement in no-HS epilepsy across two independent cohorts, with the most robust effects localized to the central subnucleus. Extending this structural phenotype, multimodal [^18^F]FEPPA PET/MR imaging contextualizes volume alterations with glial state. While a higher degree of gliosis is generally associated with atrophy, our exploratory findings are consistent with the idea that psychiatric risk is highest in individuals exhibiting greater gliosis-related enlargement, possibly reflecting a distinct state of active neuroimmune dysfunction.

These results support a shift away from treating amygdala enlargement as a static imaging phenotype and towards interpreting volumetric changes in focal epilepsy through their molecular and cellular composition. In patients with no-HS, diffuse gliosis rather than focal neuronal loss may underlie both poor surgical outcomes and elevated psychiatric risk, suggesting that glial state is a critical, yet under-recognized, dimension of disease heterogeneity.

From a translational perspective, multimodal imaging that integrates structural MRI with molecular markers of gliosis may enable biologically informed stratification of patients prior to invasive intervention. While our study is not designed to test therapeutic efficacy, the identification of a glia-associated amygdala subtype raises the testable hypothesis that a subset of patients with focal epilepsy and elevated psychiatric burden may receive a dual benefit from immunomodulatory interventions rather than standard anti-seizure or antidepressant approaches alone.

More broadly, these findings motivate future longitudinal studies combining continuous psychiatric phenotyping with *in vivo* measures of neuroinflammation to clarify how immune-related brain states contribute to affective symptoms across neurological disease. For example, anhedonia has been posited as a transdiagnostic immune-related phenotype of psychiatric illness,^52,53^ and patients with epilepsy and inflammation may be similarly predisposed. Amygdala gliosis may represent an underexplored translational target for understanding and ultimately mitigating the psychiatric burden associated with pharmacoresistant focal epilepsy.

## Supplementary Material

fcag394_Supplementary_Data

## Data Availability

Data supporting the discovery findings of this study are publicly accessible via GitHub (https://github.com/data-hcp). Data supporting the mechanistic findings of this study and analysis code are available from the corresponding author upon reasonable request and IRB approval.
