# Supplementary material for: Amygdala volume and psychiatric burden in focal epilepsy: PET evidence for a glial substrate
Source: Brain Commun. 2026 Oct 16;8(5):fcag394. doi: 10.1093/braincomms/fcag394 (PMC13711669; doi:10.1093/braincomms/fcag394)
Supplement: Supplementary file 1 [file fcag394_supplementary_data.pdf]

# Supplementary material

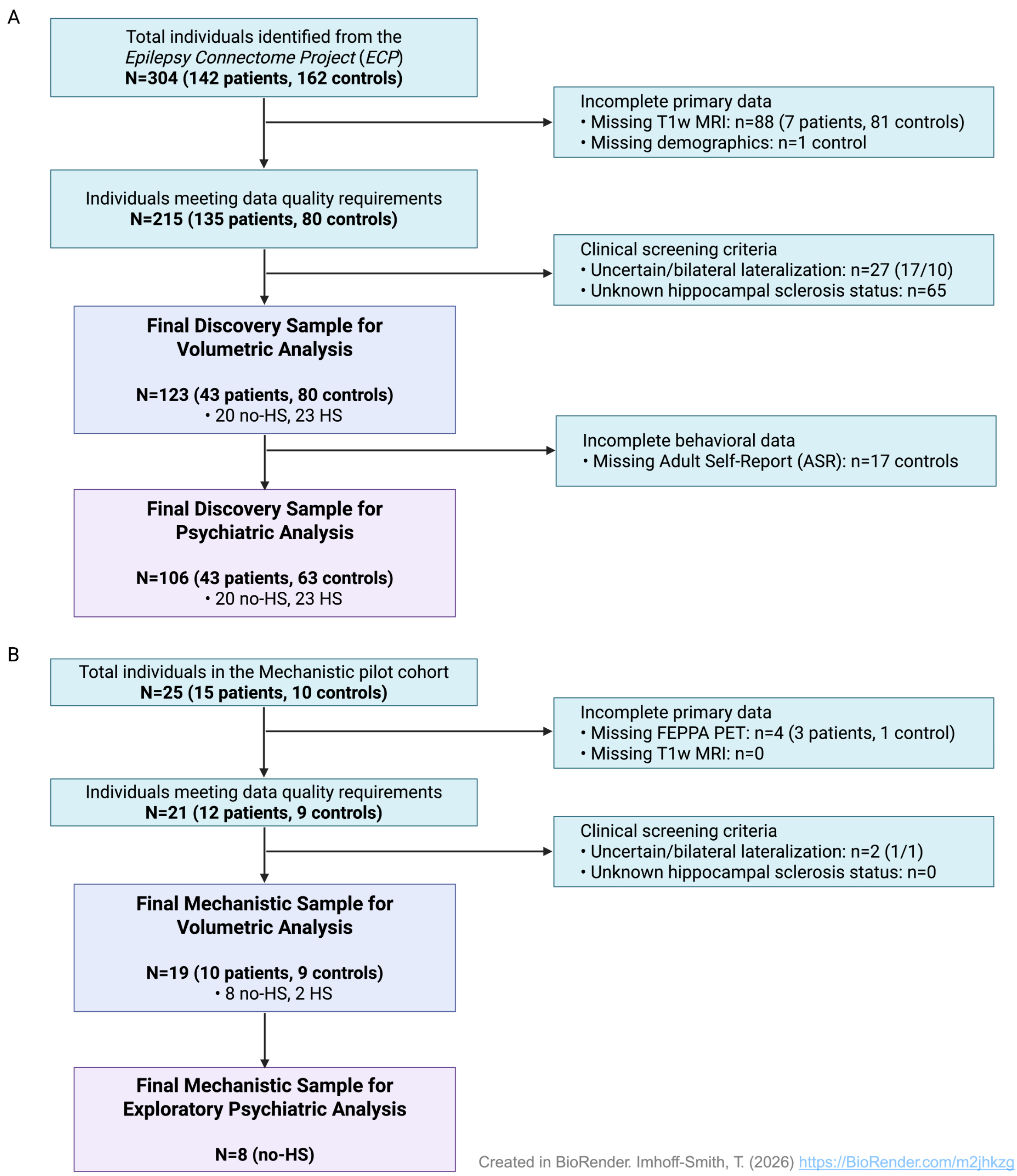


**Supplementary Figure 1. Participant screening and eligibility.** (**A**) Sequential screening procedures used to identify the Discovery sample for volumetric (N=123) and psychiatric (N=106) analyses from the Epilepsy Connectome Project repository. (**B**) Screening and exclusion procedures for participants from the Mechanistic pilot cohort. Created in BioRender. Imhoff-Smith, T. (2026). https://BioRender.com/m2jhkzg.
Alt text: Two sequential flowcharts detailing the screening, eligibility, and exclusion procedures for the Discovery sample and the Mechanistic pilot cohort, resulting in the final participant counts for the volumetric and psychiatric analyses.

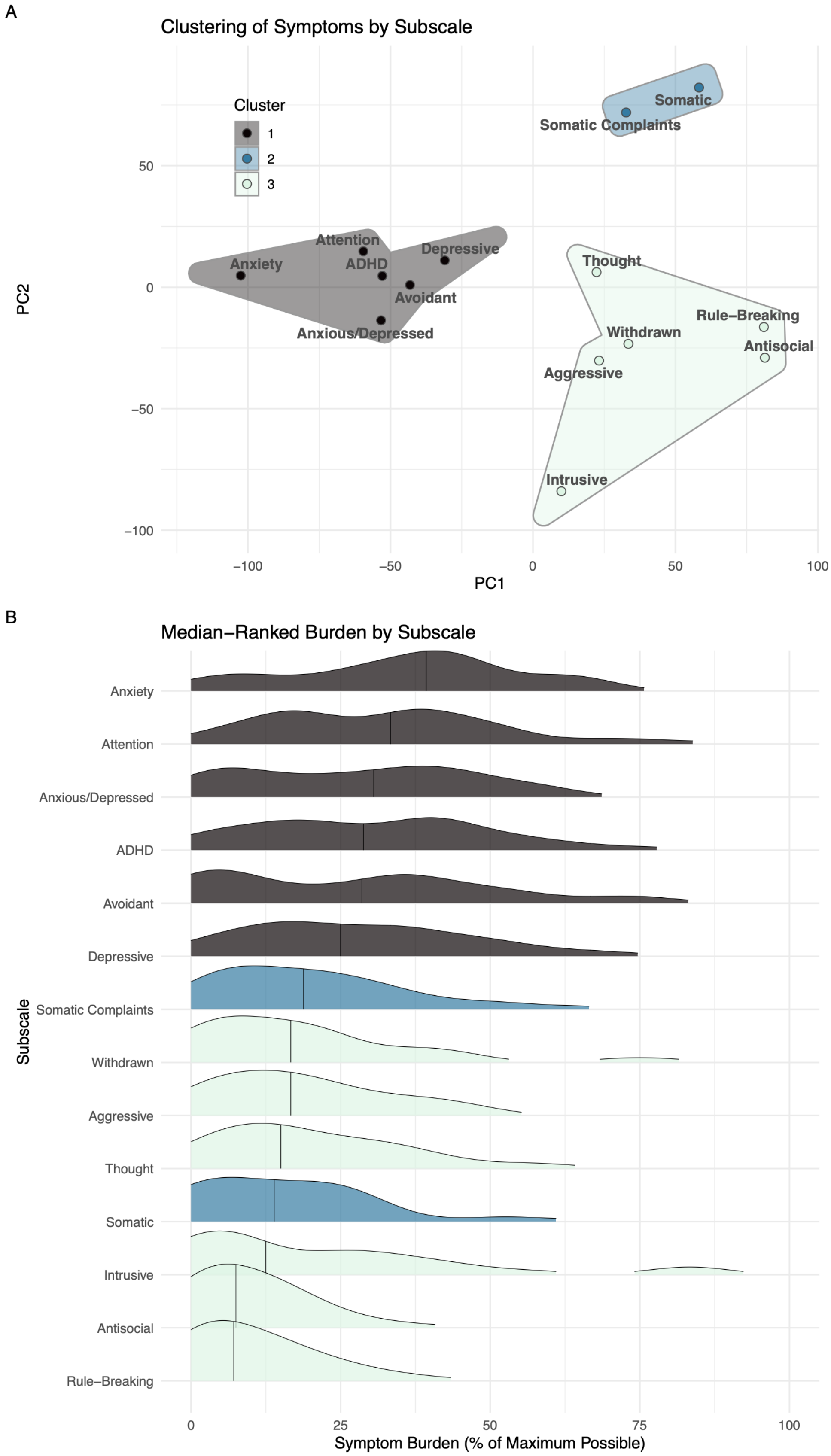


**Supplementary Figure 2. Adult Self-Report subscale clusters and burden prevalence.** (A) Two-dimensional projection of the principal component analysis space based on Percentage of

Maximum Possible (POMP; 0-100% of the theoretical maximum for each scale) scores from patients in the Discovery cohort (N=20 no-HS and 20 HS). Note that three patients with HS were excluded from this symptom domain characterization due to missing scores for the Antisocial subscale. Subscales in closer proximity indicate higher degree of symptom co-occurrence within the sample. Colors represent clusters identified via k-means clustering with 3 centroids. Each data point within those clusters represents an individual Adult Self-Report subscale (e.g., anxiety, attention, depression) positioned according to its loading on the first two principal components. The predominant cluster (left) suggests frequent co-occurrence of depression, anxiety, and attention-related scales. (**B**) Ridgeline density plots illustrate the prevalence of symptoms from each subscale across the sample, ordered from highest to lowest median. The x-axis represents the POMP score. Vertical lines indicate the sample median for each subscale. Colors correspond to the clusters identified in **A**, demonstrating that anxiety, depression, and attention cluster also represents the greatest overall magnitude of burden in this sample.

Alt text: A two panel figure summarizing symptom clusters and burden. Panel A is a two dimensional principal component analysis plot grouping individual symptom subscales into three clusters. The Anxiety, Attention, Anxious Depressed, ADHD, Avoidant, and Depressive subscales form cluster one. Panel B shows ridgeline density plots for each subscale sorted from top down by the sample medians. The density plots are shaded by cluster, showing that cluster one is formed by the subscales with the most elevated symptoms.

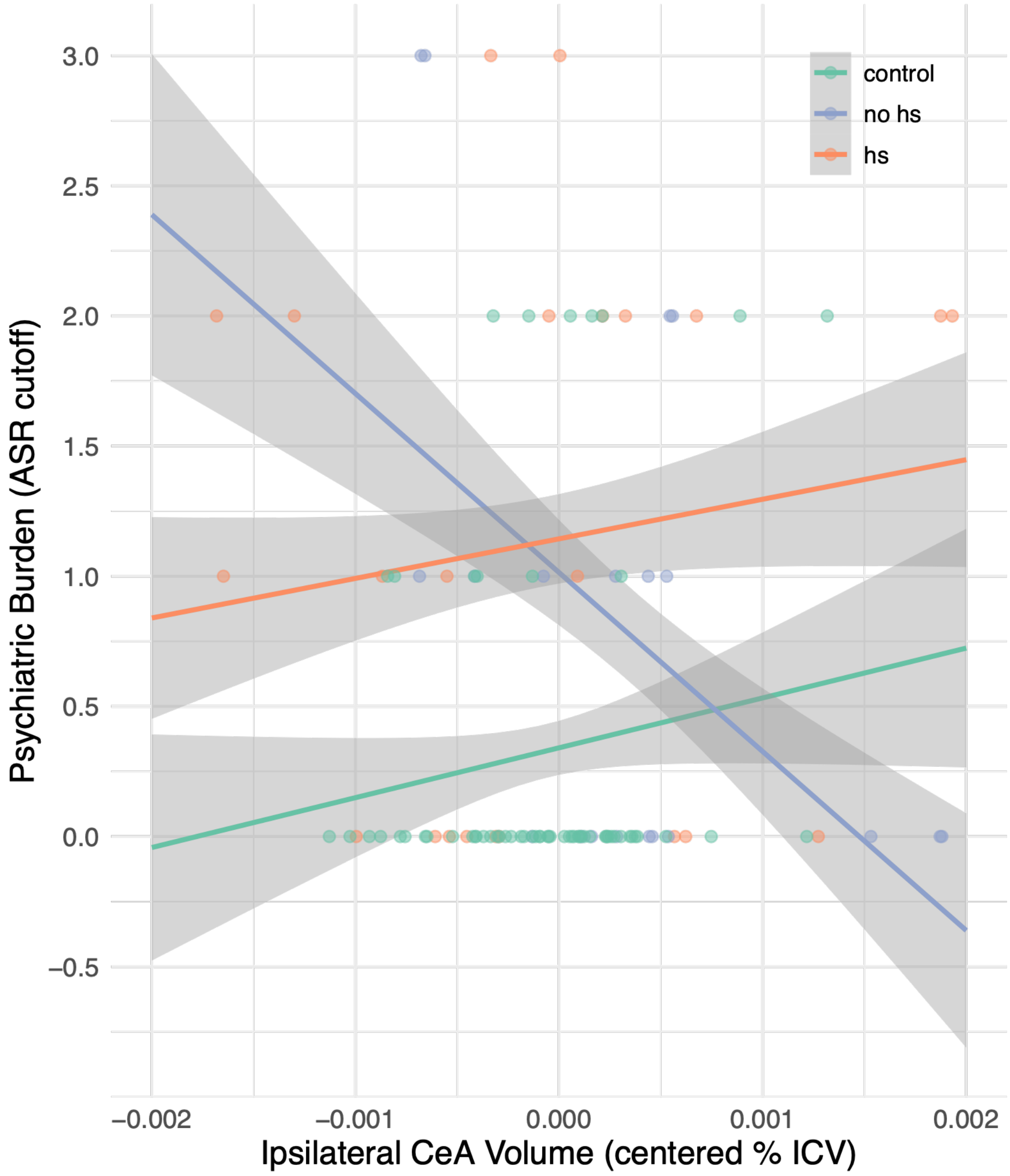


**Supplementary Figure 3. Validation of the composite psychiatric burden index in the Discovery cohort.** Illustration of the interaction between amygdala volume and the composite index of psychiatric burden derived from dichotomized T-scores in the Discovery cohort. Reduced central amygdala (CeA) volume is associated with increased psychiatric burden in the no-HS group (N=20) relative to both HS (t(98)=-2.73, p=0.007, padj=0.010; N=23) and controls (t(98)=-2.62, p=0.010, padj=0.010; N=63). This discrete composite reproduces the significant ‘group’ by ‘volume’ effect observed in the primary model of depressive symptoms (**Fig. 2A**), confirming that the discrete, cumulative index is sufficiently sensitive to capture the same underlying biobehavioral relationship as the continuous measure. Multivariable linear model results are displayed with raw or mean-centered (x-axis) data points where each data point represents independent measures from a single patient.

Alt text: A scatter plot showing a negative association between central amygdala volume and the discrete cumulative index of psychiatric burden in patients without hippocampal sclerosis.
